# Supplementary material for: Maytenus octogona Superoxide Scavenging and Anti-Inflammatory Caspase-1 Inhibition Study Using Cyclic Voltammetry and Computational Docking Techniques
Source: Int J Mol Sci. 2023 Jun 28;24(13):10750. doi: 10.3390/ijms241310750 (PMC10341947; doi:10.3390/ijms241310750)
Supplement: Supplementary file 1 [file ijms-24-10750-s001.zip › ijms-2477018-supplementary.pdf]

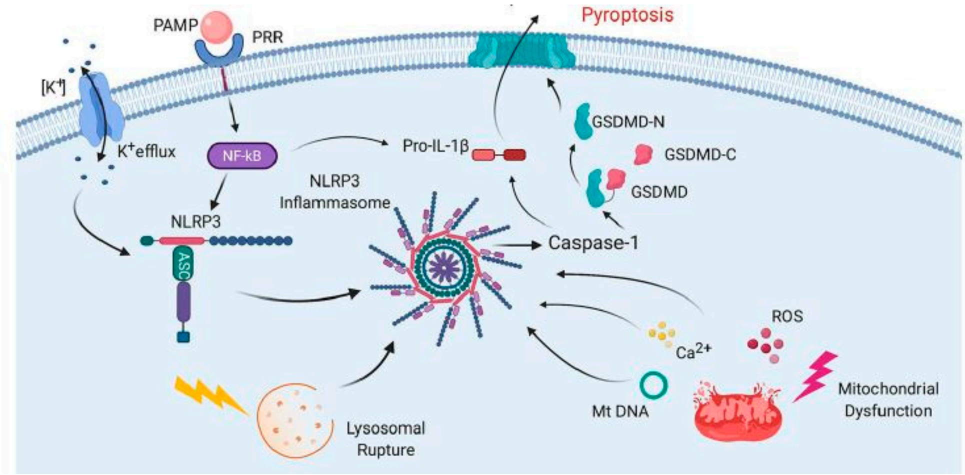

Figure S1. Mechanisms of inflammasome activation. The activation in general relies on two signals. First, a Pathogen-Associated Molecular Patterns (PAMP) binds to a pattern recognition receptors (PRR), resulting in synthesis of NLRP3 and pro-IL-1β. Then a second signal causes inflammasome assembly, leading to the activation of Caspase-1, and processing pro-IL-1β into IL-1β and pyroptosis. The second signal may come from a variety of pathways, including K^+ efflux, lysosomal rupture or mitochondrial dysfunction. This results in the release of ROS, Ca^{2+} and mitochondrial DNA (mtDNA), all of which have been shown to activate the inflammasome. Pyroptosis occurs as a result of Caspase-1-mediated cleavage of GSDM-D at the linker region of GSDM-D. Following GSDM-D cleavage, the amino terminus of GSDM-D (GSDM-D-N) forms a non-selective pore at the cell membrane through which IL-1β is then released. (From [6], with permission).

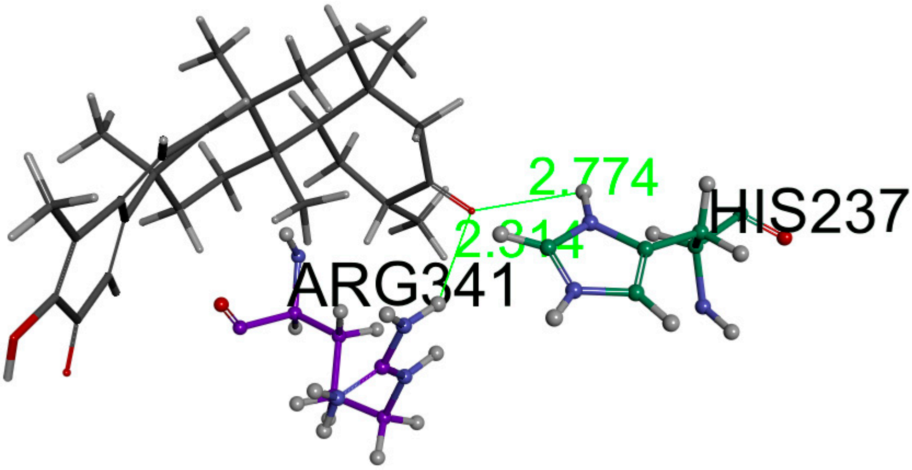

Figure S2. Atomic display of tingenone pose 1 related to Figure 5. The non methide-quinone carbonyl establishes H-bonds to the donor imidazole ring of His237 and the donor guanidino group of Arg341, 2.774 Å and 2.314 Å, respectively.

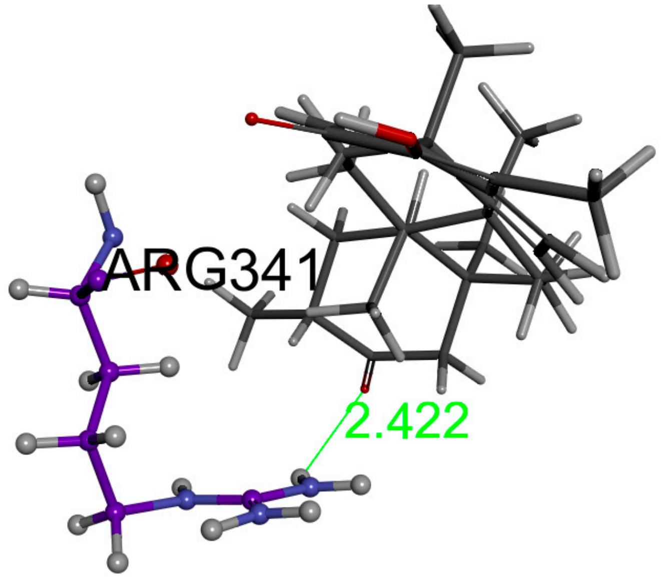

Figure S3. Atomic display of tingenone pose 5 related to Figure 7. The non methide-quinone carbonyl establishes a H-bond with the guanidino group of Arg341, 2.422Å.

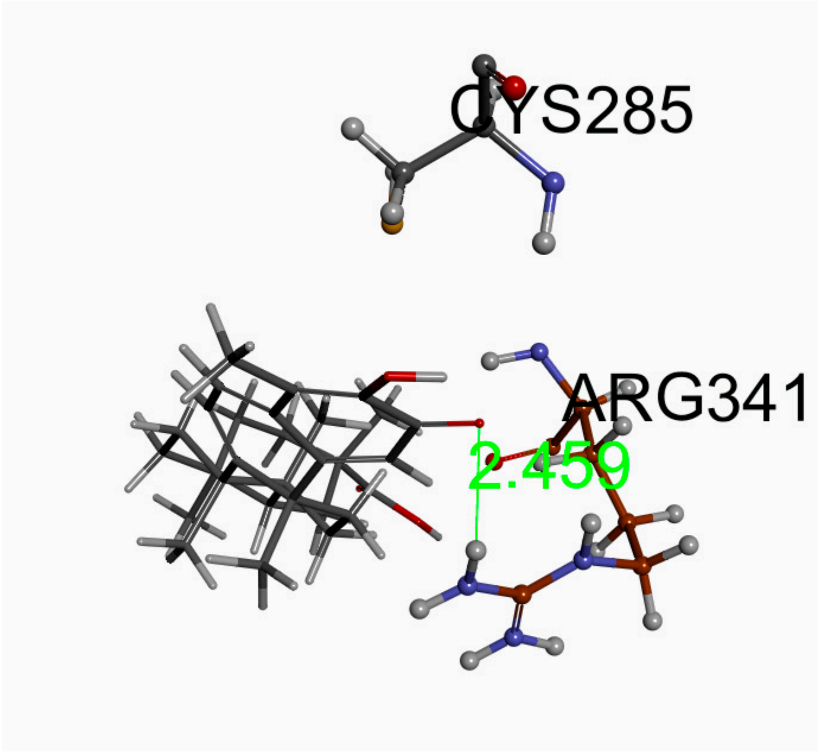

Figure S4. Atomic display of pristimerin docking pose 4 related to Figure 9. The non methide-quinone carbonyl establishes with donor guanidino a H-bond Arg341, 2.459 Å. Although Cys285 is close to pristimerin methide-quinone ring, a π-sulfur interaction is not obvious.
